# Obituary—when somebody close to us dies, part of our future dies with them: John A. Fixsen, MChir, FRCS

**DOI:** 10.1186/s13018-014-0111-6

**Published:** 2014-11-20

**Authors:** Nicola Maffulli

**Affiliations:** Department of Musculoskeletal Disorders, University of Salerno, 89100 Salerno, Italy; Centre for Sport and Exercise Medicine, Queen Mary University of London, London, E1 4DG UK

It is always difficult to write about individuals who are close to you and who forged you in your formative years.

In August 2014, Mr. John A. Fixsen, MChir, FRCS, Consultant Emeritus in Paediatric Orthopaedics at The Hospital for Sick Children, Great Ormond Street Hospital, London, and Consultant Orthopaedic Surgeon Emeritus at St Bartholomew’s Hospital, London, died in his sleep.

I knew of Mr. Fixsen, but I met Mr. Fixsen in 1987, when I was appointed a Lecturer at the Institute of Child Health, and Honorary Registrar in his department. At the time, I still did not understand much of the British system of training, with the multitude of grades for junior doctors, but I knew one thing: it was jolly good!

Mr. Fixsen took me under his wing and pushed me to love Paediatric Orthopaedics. My job was mainly academic, and I was leading a team of nine people to study the effects of intensive training in young athletes, the Training of Young Athletes (TOYA) study. I learnt that he was ‘JAF’ when his trainees spoke of him and soon realized that he had the most encyclopaedic knowledge of orthopaedics that I had ever encountered. After the afternoon ward round, which in reality would be in the early evening, the whole team would convene at the Lamb, the great pub in Lambs Conduit Street, to talk about life, and for John to have two half pints of bitter. There, at one stage, he revealed that he had been fluent in Russian. As he had been a conscript in the Cold War era, one of us asked him whether he had been a spy. He never answered!

I spent 4 years at Great Ormond Street, and JAF taught me about Paediatric Orthopaedics. His vision was, and remains, unique. He was, at one stage, the foremost paediatric orthopod in the English speaking world, but the quirk of his appointment, whereby he continued to undertake adult orthopaedic practice until he retired, allowed him to continue to follow up his patients until maturity, and in this way to really see whether what he had done stood the test of time. I became part of the team and, through him, learnt the inner working of Great Ormond Street Hospital. What a great place it was.

JAF guided me. His empathy was unique, his diagnostic skill unsurpassed, his technical surgical abilities left me thinking ‘No matter how many times I shall do that, I shall never be that good’. I left GOS to continue my clinical training, and my time off was spent at GOS, doing clinics with him and assisting in theatre. After my Ph.D. in Paediatric Sports Medicine, JAF told me ‘Do you realise that you have done enough research to do a Master of Surgery?’, and so I ended up writing a thesis in limb lengthening.

My Senior Registrar appointment was in Aberdeen, and I always kept JAF abreast of what I was doing. He was genuinely interested, and when I left the UK to become an Associate Professor and Consultant in Hong Kong, JAF visited me. I wished to return to London, at GOS, and we started talking. At the time, he asked me to stop addressing him as Mr. Fixsen, and we came to first name terms. What an honour!

JAF told me that he wished to retire in 1997 and that anybody appointed at GOS would have shadowed him for 1 year, until his retirement, a great opportunity to slip into the environment at GOS with less fear and problems. I went to GOS on several occasions: John knew that I was determined to pursue an academic career, and he pushed jolly hard to create a Senior Lectureship. The powers that be did not budge, and a Consultant job was instead advertised. I decided, crying inside, not to apply.

The rest is history. Great men have taken on John’s legacy at GOS, and he retired, as promised, in May 1997. At the time, a retirement scientific meeting was held at Bart’s. I had been the first of John’s advanced training fellows and was asked to give a talk on behalf of all of them. We laughed and we rejoiced, and I always kept in touch. After announcing his retirement, John spent significant amount of his ‘free’ time in Afghanistan, in Red Cross hospitals, continuing to do what he always did: take care of sick children and adults.

I always informed him of my moves. I became first the Professor of Trauma and Orthopaedic Surgery at Keele University, and then the Professor of Sports and Exercise Medicine in London: John came to my Inaugural Lecture in November 2010 at Queen Mary University of London, and we spoke about how I was still seeing young athletes, doing paediatric trauma, and taking care of some neuromuscular conditions in children. I told him how I was applying the principles that he taught me to neuromuscular conditions in adults, and he smiled.

We met at the board meetings of the Journal of Bone and Joint Surgery, British Volume on a regular basis. I will always remember John for being able to put everything into perspective and for the ability to talk to anybody about everything. Above all, I will remember how John opened doors for me, and he made sure that a little Italian doctor transplanted in London was put on par with the local public school boys and was never allowed to get it wrong.

Thank you, John. You helped me to shape my future: you will always be in my thoughts.

